# Quantitative Galactose Colorimetric Competitive Assay Based on Galactose Dehydrogenase and Plasmonic Gold Nanostars

**DOI:** 10.3390/bios13110965

**Published:** 2023-11-01

**Authors:** Tozivepi Aaron Munyayi, Danielle Wingrove Mulder, Engela Helena Conradie, Frans Johannes Smit, Barend Christiaan Vorster

**Affiliations:** 1 North-West University, Potchefstroom Campus, Potchefstroom 2531, South Africa; 2 Center for Human Metabolomics, North-West University Potchefstroom Campus, Potchefstroom 2531, South Africa; danielle.mulder53@gmail.com (D.W.M.); elne.conradie@nwu.ac.za (E.H.C.); chris.vorster@nwu.ac.za (B.C.V.); 3 Research Focus Area for Chemical Resource Beneficiation, North-West University, Potchefstroom 2520, South Africa; frans.smit@nwu.ac.za

**Keywords:** gold nanostars, enzymatic, immobilized, plasmonic, colorimetric, detection solution

## Abstract

We describe a competitive colorimetric assay that enables rapid and sensitive detection of galactose and reduced nicotinamide adenine dinucleotide (NADH) via colorimetric readouts and demonstrate its usefulness for monitoring NAD+-driven enzymatic reactions. We present a sensitive plasmonic sensing approach for assessing galactose concentration and the presence of NADH using galactose dehydrogenase-immobilized gold nanostars (AuNS-PVP-GalDH). The AuNS-PVP-GalDH assay remains turquoise blue in the absence of galactose and NADH; however, as galactose and NADH concentrations grow, the reaction well color changes to a characteristic red color in the presence of an alkaline environment and a metal ion catalyst (detection solution). As a result, when galactose is sensed in the presence of H_2_O_2_, the colored response of the AuNS-PVP-GalDH assay transforms from turquoise blue to light pink, and then to wine red in a concentration-dependent manner discernible to the human eye. This competitive AuNS-PVP-GalDH assay could be a viable analytical tool for rapid and convenient galactose quantification in resource-limited areas.

## 1. Introduction

Gold nanoparticles (AuNPs), particularly gold nanostars (AuNSs), have gained considerable attention in diagnostics due to their unique, size-dependent characteristics, biocompatibility, and the simplicity of their chemical manipulation [1,2,3,4,5]. AuNSs display polarization-dependent scattering and absorption with dual spectral peaks (transverse and longitudinal) that are highly sensitive to protruding tip eccentricity changes [6,7,8,9,10]. Plasmonic AuNSs have extraordinarily high extinction coefficients since their surface plasmon resonance is strongly impacted by their size, shape, and morphology [11,12]. AuNSs are extremely sensitive to environmental perturbations, resulting in evident morphological, spectrometric, and colorimetric changes [3,13]. These excellent properties could pave the way for the development of nanosensors with simple plasmonic-absorbance-based readouts [14].

Nicotinamide adenine dinucleotide (NAD+) is a redox coenzyme that is essential for energy metabolism [15]. As a redox carrier, NAD+ accepts a hydride ion and shuttles electrons from metabolic activities to generate its reduced form, NADH [16]. NADH can donate one or two electrons depending on the substrate’s Gibbs free energy [17]. Several NAD+/NADH-redox-coupled sensing approaches have been developed, with the majority requiring sophisticated processes and specialist personnel to interpret the results [18,19,20,21,22]. Traditional colorimetric methods for detecting NAD/NADH are based on the color reaction of organic dyes, which are quickly oxidized by O_2_ [23,24]. As a result, establishing a reliable NAD/NADH detection technique free of chromogenic substrates is crucial. Plasmonic-based biosensing has been widely employed for on-site or in-field solutions to complement traditional diagnostic procedures due to its versatility, low cost, simple result interpretation, and robust reaction times [25,26]. As a result, NAD+/NADH redox paired with plasmonic colorimetric sensing has emerged as a feasible alternative to existing methods [27,28]. 

Many plasmonic sensors have been constructed by monitoring the resonant electromagnetic field and being sensitive to changes in the refractive index of the medium around nanoparticles after target molecule (NAD/NADH) binding [29]. Liang et al.(2015) recently disclosed a paper-based system for quick and sensitive room-temperature measurement of NADH using a colorimetric readout [27]. In their work, NADH was found to limit AuNP dissolution by reducing Au^3+^ to Au^+^, resulting in a red color that became deeper with increasing NADH concentrations. Using a similar approach, Baymiller et al. (2017) explored NADH’s ability to produce nanoparticles and discovered that this coenzyme alone is adequate to convert Au^3+^ ions to gold nanoparticles in vitro [30]. At room temperature, this method creates uniformly dispersed spherical plasmonic nanoparticles of varying sizes almost instantly, with NADH concentration regulating both the rate of synthesis and the tonality of the produced gold nanoparticles (AuNPs). Using p-aminophenol (pAP)-catalyzed and NADH-mediated production of silver nanoparticles (AgNPs), Liu et al. (2021) developed a light-controlled colorimetric assay for the quantification and detection of nitroreductase (NTR) [31]. The hydrolysis product of p-nitrophenol from NTR in the presence of NADH can be employed as a catalyst for the reduction of Ag^+^ by NADH under light, providing NTR concentration-dependent colorimetric signals. Furthermore, NAD n NADH are widely employed to quantify substrate-to-product conversion directly or indirectly [32,33]. In contrast to NADH sensing, hydrogen peroxide (H_2_O_2_) plasmonic sensing has been intensively researched in recent years because H_2_O_2_ sensing is robust, leading to colorimetric and morphological alterations in nanoparticles [34,35,36]. When nanoparticles are exposed to H_2_O_2_, they undergo catalytic dissolution, resulting in a decrease in the nanoparticle absorption band with increasing H_2_O_2_ concentration [37]. Overall, plasmonic biosensors generate colorimetric signals primarily as a result of the aggregation/dispersion degree caused by the addition of Au^3+^ solution or through the etching/growth of pre-made nanoparticles in the presence of an alkaline environment and a metal ion catalyst (detection solution) [34,38].

In this study, rather than measuring NAD/NADH, we investigated their suitability as redox agents for gold nanostar shape alteration in colorimetric quantification of varied galactose concentrations. We developed a nanoplasmonic sensor for quantitatively detecting NADH and galactose in diverse matrices using galactose dehydrogenase (GalDH)-NAD+-co-immobilized AuNS. This paper presents an integration of H_2_O_2_-induced surface etching of AuNS- and NADH-driven biocatalytic growth of AuNPs in the presence of a detection solution to yield morphological and colorimetric changes. This colorimetric detection system takes advantage of NADH inhibiting AuNS dissolution while H_2_O_2_ induces AuNS dissolution, cyclic oxidation of NADH, and biocatalytic growth of AuNPs [27,39]. We also used redox chemistry to investigate how the detection solution affects colorimetric signal generation from both experimental and theoretical perspectives. As a result, this assay has significant potential for in-field analytes and inhibitor screening with the naked eye, with a mechanism that is most likely transferable to other NAD+-dependent enzymes. 

## 2. Materials and Methods

All materials were bought from Sigma-Aldrich unless stated otherwise. The chemicals used were sodium hydroxide, 4-(2-hydroxyethyl)-1-piperazineethanesulfonic acid (HEPES, C_8_H_18_N_2_O_4_S), gold (III) chloride trihydrate (HAuCl_4_·xH_2_O), silver nitrate (AgNO_3_), sodium hydroxide (NaOH), 3′30–dithiobis sulfosuccinimidyl propionate (DTSSP, C_14_H_14_N_2_O_14_S_4_Na_2_), galactose (C_6_H_12_O_6_), hydrogen peroxide (H_2_O_2_), galactose dehydrogenase (GalDH), nicotinamide adenine dinucleotide hydrate (NAD, C_21_H_27_N_7_O_14_P_2_·xH_2_O), Tris buffer (NH_2_C(CH_2_OH)_3_, and polyvinylpyrrolidone (PVP) 10,000. Synthetic dry whole blood was purchased form Thistle QA, Johannesburg, South Africa. The materials used were sterile 5 mL screw cap tubes (Ascendis Medical), carbon mesh copper grids (Agar Scientific, Rotherham, UK), and clear, flat-bottomed 96-well plates (Corning).

### 2.1. Instrumentation and Characterization

Scanning electron microscopy and energy-dispersive x-ray spectroscopy (SEM-EDS, Bruker, Billerica, MA, USA) were used to assess the chemical compositions of the samples qualitatively. Inductively coupled plasma mass spectrometry (ICP-MS, Spectro AMETEK, Inc., Kleve, Germany) was used to determine the elemental contents in the samples. SEM-EDS and ICP-MS were utilized to precisely quantify and confirm the chemical composition of the samples. 

The gold nanostar and bioconjugate spectrum scanning (400–900 nm) was carried out utilizing 96-well plates and the HT Synergy (BioTEK) microplate reader (Agilent Technologies, Santa Clara, CA, USA).

High-resolution transmission electron microscopy (HR-TEM) was carried out using a Tecnai F20 transmission electron microscope (JEOL, Freising, Bavaria, Germany). The capped gold nanostar samples were imaged after being spotted onto copper grids (Agar Scientific) and air-dried. In contrast, the gold nanostar bioconjugates required 1% silver nitrate solution staining prior to copper grid spotting, air drying, and imaging for protein visualization [40].

ImageJ software (bundled with 64-bit Java 1.8.0_172, University of Wisconsin at Madison, Madison, WI, USA) was used to estimate particle core diameters and arm dimensions by averaging a total count of one hundred capped gold nanostars and one hundred gold nanostar bioconjugates in different HR-TEM images.

All nuclear magnetic resonance (NMR) experiments at 500 MHz were performed with a Bruker Avance III HD (NMR) spectrometer equipped with a triple-resonance inverse (TXI) 1H (15N, 13C) probe head (Bruker, Billerica, MA, USA). This was used to confirm the gold nanostars’ successful capping and protein immobilization.

The capped gold nanostars and gold nanostar bioconjugates were electrophoresed in agarose gel using a Baygene BG-power Vacutec electrophoresis gel apparatus (Vacutec, Johannesburg, South Africa). To confirm the molecules based on their size and electrical charge, electrophoresis was performed at pH 8 with 0.5% agarose and 0.5 × Tris borate EDTA buffer (TBE buffer). The samples were made by combining 32 µL of nanostars with 4 µL of 80% glycerol and running them at 40 V for 45 min. The gold nanostar bioconjugate gels were stained for 4 h with 25 mL of 0.25% (*w*/*v*) Coomassie blue and then rinsed with a destaining solution (90% *w*/*v* isopropanol and 10% *w*/*v* glacial acetic acid), revealing the protein bands. Gels were stored in ddH_2_O until photos were recorded and uploaded to a computer.

### 2.2. Methods

#### 2.2.1. Preparation of Gold Nanostars

The gold nanostars (AuNSs) were synthesized using the modified HEPES buffer method described and characterized by our laboratory [41,42]. Succinctly, 2 mL of 100 mM HEPES buffer (pH 7.4) was added to 3 mL of deionized water (Millipore, Temecula, CA, USA, 18.2 ΩM·cm^−1^), followed by 20 µL of 50 mM gold (III) chloride trihydrate (HAuCl_4_·xH_2_O) and 4 µL of 1 mM silver nitrate (AgNO_3_). The 5 mL screw cap was mixed via end-to-end tube inversion and incubated for 25 min at room temperature until the solution turned turquoise blue. The nanostars were then capped with 600 µL of 2.5 mM PVP. After inverting the tube several times, it was left to stand at room temperature for 1 h. The capped AuNS sample suspension was cleaned twice via centrifugation for 35 min at 2170× *g* and resuspended in 500 μL of ddH_2_O.

#### 2.2.2. Preparation of Gold Nanostar–Galactose Dehydrogenase Bioconjugate

The capped AuNS sample suspension was cleaned twice via centrifugation for 35 min at 2170× *g* and resuspended in 500 μL of 100 mM HEPES (pH 6.9). Four batches of 500 μL each were then combined, making for a total volume of 2 mL, and 100 μL of 5 mM DTSSP was then added to the solution, followed by 100 μL of 8 mM NAD+ and 150 μL of GalDH 2 mg/mL. The solution was incubated at 4 °C for 2 h to allow enzyme and coenzyme conjugation and immobilization. This co-immobilization was carried out to improve enzyme stability, activity, and selectivity or specificity [43,44,45]. Finally, the co-immobilized AuNS (AuNS-PVP-GalDH) was cleaned twice via centrifugation at 2170× *g* for 35 min and then resuspended in 500 μL of ddH_2_O (Millipore, 18.2 ΩM·cm^−1^) and stored at 4 °C.

#### 2.2.3. Gold Nanostar–Galactose Dehydrogenase Biosensor Colorimetric Assay

The AuNS-PVP-GalDH plasmonic colorimetric tests were performed using galactose as an analyte and different H_2_O_2_ concentrations (0 mM, 0.05 mM, 0.10 mM, 0.15 mM, and 0.20 mM). Five distinct experiments were performed (as summarized in Table 1), with everything kept constant and varied H_2_O_2_ concentrations. The reagents were pipette-mixed in water to a final volume of 200 μL in the following order: Following the addition of 15 μL of 10 mM Tris buffer (pH 8.4) and 20 μL of AuNS-PVP-GalDH to each of the five experiments, different volumes (0 μL, 5 μL, 10 μL, 15 μL, and 20 μL per experiment) of 2 mM galactose were added and pipette-mixed into the assays. The experiments were incubated for 10 min at 37 °C before adding (0 μL, 5 μL, 10 μL, 15 μL, and 20 μL) each H_2_O_2_ concentration employed. The assays were then incubated for 5 min at 37 °C before adding the detection solution (2 μL 10 mM AgNO_3_ + 15 μL 150 mM NaOH). Finally, the assays in each experiment were incubated for 2 min at 37 °C before obtaining UV-Vis spectral readings and colorimetric signals.

A total of 5 mg of synthetic whole blood (powder) was dissolved in 1.5 mL of ddH_2_O, and end-to-end inversion was performed to ensure appropriate powder dissolution. In 5 mL screw cap tubes, several galactose solutions (2 mM, 4 mM, 6 mM, and 8 mM) were produced and nitrogen-dried for 24 h to reduce contamination and overdilution of the matrix. To guarantee adequate spiking, the dried galactose solution screw cap tubes were diluted with 500 µL of synthetic blood and inverted end-to-end. After spiking the synthetic blood matrix with galactose, the samples were serially diluted 1000 times for easy spectrophotometric reading acquisition [46]. The specificity and efficacy of the AuNS-PVP-GalDH assay were determined using spiked synthetic whole blood with various amounts of galactose. The detection of galactose was tested spectrophotometrically and visually by detecting color changes in the solutions after substituting galactose with synthetic-blood-spiked galactose, as stated above. All experiments were carried out in triplicate.

##### The Influence of the Detection Solution on the AuNS-PVP-GalDH Biosensor

The alkaline environment and metal ion catalysts’ effects on the AuNS-PVP-GalDH biosensor in the aqueous matrix were examined. Furthermore, by employing different gold nanoparticle sizes, gold nanoparticle nucleation or growth and colorimetric signal generation induced by pH fluctuations over time and metal ion catalysts were investigated [47].

## 3. Results

**Figure 1:**
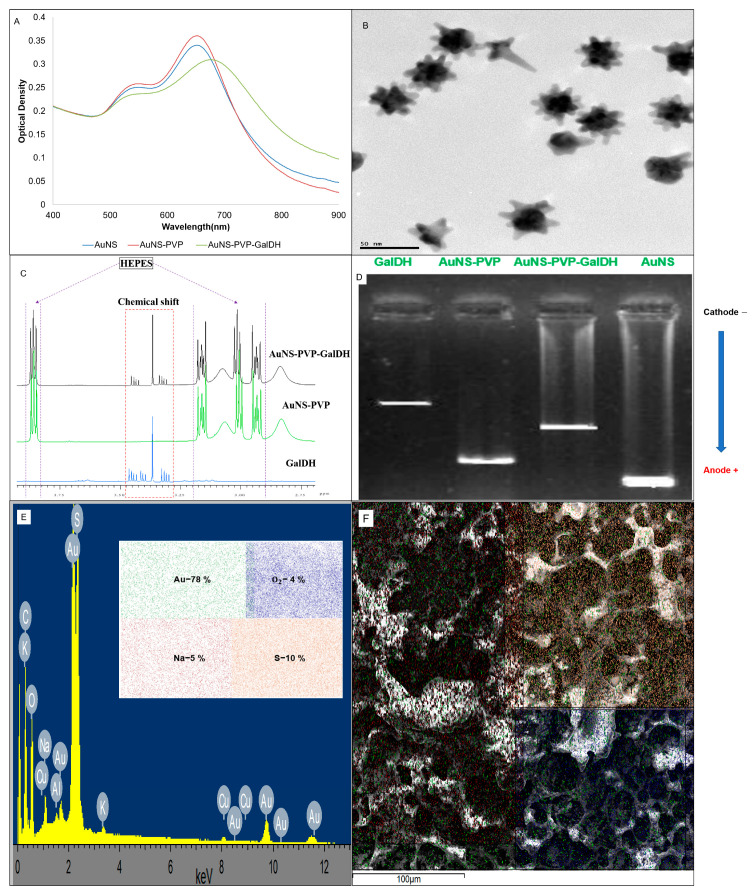
AuNS-PVP-GalDH bioconjugate’s (**A**) UV-Vis spectra profiles; (**B**) HR-TEM profiles; (**C**) 1H-NMR spectra profiles; (**D**) electrophoretic profiles; (**E**) EDS spectra; and (**F**) SEM-EDS elemental mapping profiles.

Figure 1 depicts the UV-vis spectral scans, ^1^H-NMR spectra, HR-TEM images, and electrophoretic migration of the AuNS-PVP-GalDH bioconjugate. As shown in Figure 1A, the distinctive surface plasmon resonance (SPR) band of AuNS red-shifted after protein addition, resulting in a drop in optical density (OD)_max_ absorbance, whereas PVP capping resulted in a modest rise in OD_max_ relative to the uncapped AuNS absorption band. This is due to the interaction and orientation of the coatings on the AuNS, polydispersity, and the size of the side groups in the proteins and PVP, which cause different shifts in the AuNS absorption band. The HR-TEM images in Figure 1B demonstrate that the AuNS-PVP-GalDH complex was fairly monodispersed, with an estimated average size ranging from 28 to 30 nm and an average of eight protuberant spikes on well-developed AuNSs. Subsequently, the electrophoretic migration patterns indicate effective capping and protein bioconjugation to the AuNS rather than just being present in the sample (Figure 1C). The elemental composition and mapping images of the AuNS-PVP-GalDH bioconjugate are shown in Figure 1E,F. Furthermore, quantitative ICP-MS (results not given) and EDS data showed the presence of gold and other trace metals in the AuNS-PVP-GalDH bioconjugate.

These findings reveal that the AuNS-PVP-GalDH bioconjugates were monodispersed and heterogeneous and that the majority of nanostars had a spiky morphology primarily made up of gold (78%), sulfur (10%), and other trace elements, a result that is consistent with prior research [42].

**Figure 2:**
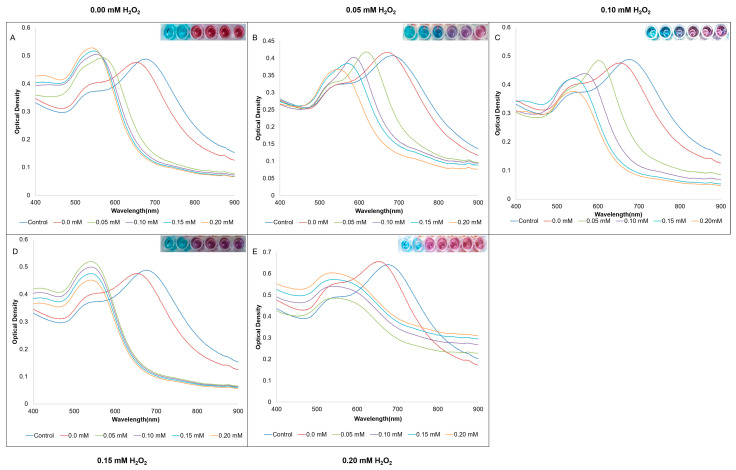
UV-Vis spectra and colorimetric profiles indicating the ability of H_2_O_2_ to induce concentration-dependent colorimetric signals for galactose detection in the AuNS-PVP-GalDH assay in the water matrix. The following are the variations in H_2_O_2_ concentrations: (**A**) 0.00 mM; (**B**) 0.05 mM; (**C**) 0.10 mM; (**D**) 0.15 mM; (**E**) 0.20 mM. The results show that concentration-dependent etching of the AuNS-PVP-GalDH assay was most visible when the H_2_O_2_ concentration was 0.05 mM, indicating that 0.05 mM was the optimal H_2_O_2_ concentration. Furthermore, the results demonstrate that H_2_O_2_ concentration affects the rate of colorimetric signal generation and the degree of longitudinal plasmon band (LPB) etching and transverse plasmon mode (TPB) growth.

Figure 2 depicts the spectrometric and colorimetric results of the AuNS-PVP-GalDH assay in various galactose and H_2_O_2_ concentrations. When utilizing the H_2_O_2_-free assay, the biocatalytic reaction occurred instantaneously, generating a distinct red color, as shown in Figure 2A. Nonetheless, the biocatalytic reaction occurred slowly in the 0.05–0.10 mM H_2_O_2_ concentration range, producing multicolorimetric readings and improved UV-Vis spectral profiles within 2 min of incubation. As shown in Figure 2D,E, H_2_O_2_ rapidly induced AuNS UV-Vis spectral profiles’ transformation into spherical AuNP profiles, and the biocatalytic reaction proceeded instantaneously in the 0.15–0.20 mM H_2_O_2_ concentration range. 

**Figure 3:**
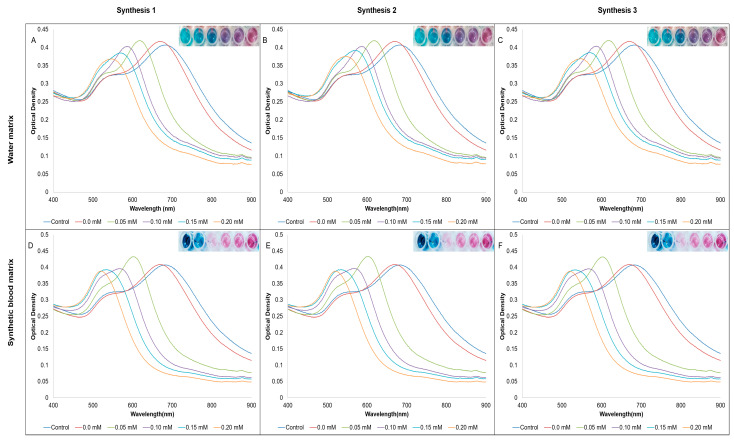
Galactose sensing with AuNS-PVP-GalDH nanosensors of various synthesis batches: UV-Vis spectra; (**A**–**C**) water matrix; and (**D**–**F**) synthetic blood matrix.

In both water and the synthetic whole-blood matrix, different synthetic batches produced nearly identical colorimetric signals in terms of tonality and slightly variable spectrophotometric readouts (Figure 3). 

**Figure 4:**
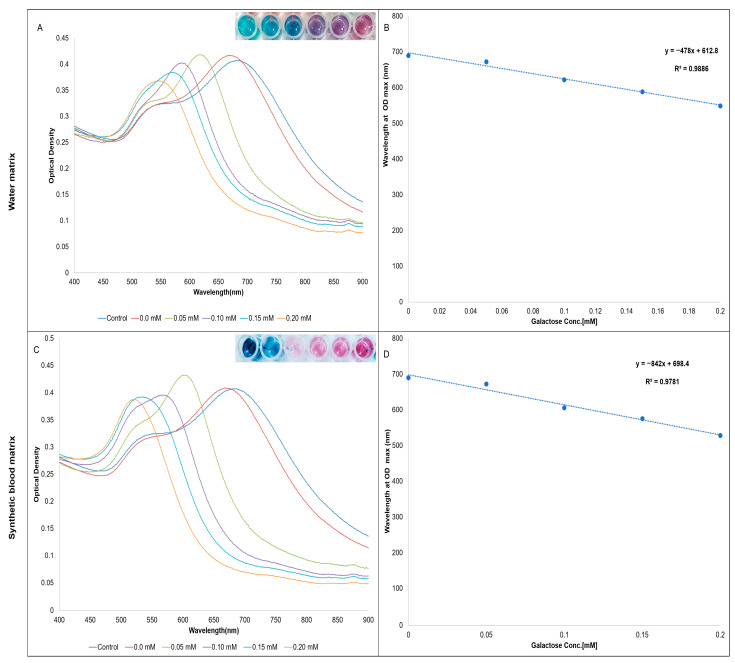
UV-Vis spectra, colorimetric profiles, and mathematical correlation profiles of galactose sensing with AuNS-PVP-GalDH nanosensor: (**A**,**B**) water matrix and synthetic blood matrix (**C**,**D**).

As a result, the colorimetric and spectrometric responses of the AuNS-PVP-GalDH bioconjugates for galactose determination were comparable. As a proof of concept, the optimized NADH-H_2_O_2_-driven assay measured various galactose concentrations in different matrices, yielding multicolorimetric and concentration-dependent spectrum signals. Figure 4 depicts the average colorimetric and spectrophotometric signals, as well as mathematical correlations, of several AuNS-PVP-GalDH biosensor syntheses in water and synthetic blood matrices.

The AuNS-PVP-GalDH spectrum before the colorimetric biocatalytic reaction exhibited two well-resolved bands centered at about 550 and 700 nm, respectively. In contrast, the LLSPR spectra (700 nm) showed a significant drop in absorbance and a strong blue shift as the galactose concentration increased, and the intensity of the TLSPR spectra (550 nm) increased. At a 0.2 mM galactose concentration, single TLSPR spectra at 550 nm were detected, demonstrating the full transition of AuNSs to quasi-spherical nanostructures. Furthermore, the subsequent changes in the AuNSs may be visualized as a progressive change in the solution color from turquoise blue to purplish blue to red pink to wine red in the water matrix and from turquoise blue to red pink to wine red in the synthetic blood matrix with good mathematical correlations (Figure 4).

**Figure 5:**
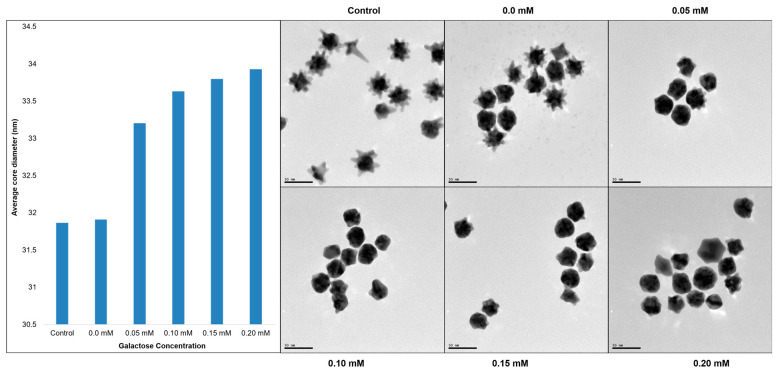
Galactose sensing with AuNS-PVP-GalDH biosensor HR-TEM images after the biorecognition reaction in the water matrix.

The HR-TEM images of the morphological alterations that occurred during the biorecognition reaction are shown in Figure 5. The nanostructures remained star-shaped in the absence of galactose. When galactose concentrations were increased, the AuNSs progressively became more quasi-spherical, with evident core diameter enlargement from approximately 30 to 34 nm. 

Overall, the colorimetric biorecognition reaction’s representative TEM images reveal a transition from a high yield of branching particles to the existence of high quasi-spherical morphologies.

**Figure 6:**
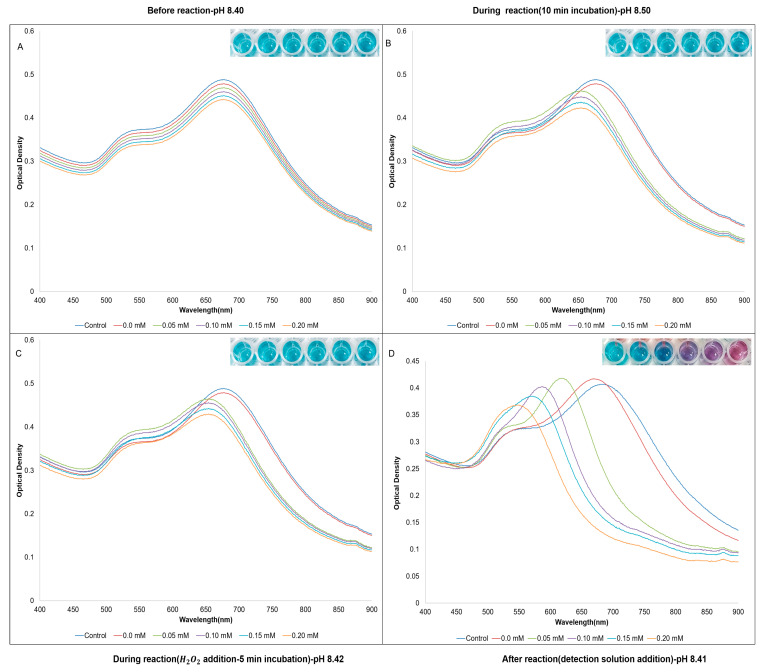
UV-Vis spectra and colorimetric profiles for pH variations during galactose detection in the water matrix using the AuNS-PVP-GalDH biosensor at different points: (**A**) Pre-reaction phase; (**B**) NADH production phase; (**C**) H_2_O_2_ introduction phase; (**D**) Colorimetric signal generation phase.

The UV-Vis spectrum changes and colorimetric signals, as well as the accompanying pH fluctuations, are depicted in Figure 5. Even in the presence of varying galactose/NADH and H_2_O_2_ concentrations, the bioassays maintained their turquoise blue color with essentially identical spectrum changes as the reaction progressed (Figure 6B,C). The bioassays only produced convincing colorimetric and spectral signals when the detection solution was added (Figure 6D), which is consistent with earlier research [34,48].

The pH increased from 8.4 to 8.5 during the 10 min incubation at 37 °C as the biosensor worked on the analyte producing NADH, and when H_2_O_2_ was added to the reaction mixture, the pH dropped to 8.42 after the 5 min incubation at 37 °C, and finally, the pH dropped to 8.41 after the addition of the detection solution and the 2 min incubation at 37 °C. Overall, even in the presence of the redox agents NADH and H_2_O_2_, there was no colorimetric signal generation in the absence of an alkaline environment and metal ion catalyst in the AuNS-PVP-GalDH biosensor.

## 4. Discussion

The HEPES-mediated seedless one-pot synthesis protocol was utilized to synthesize anisotropic AuNSs. In this approach, HEPES worked as a mild reducing, stabilizing, and shape-directing agent, resulting in heteromorphic nanostars of varying diameters (Figure 1B) [49]. Numerous studies have demonstrated that HEPES has a high affinity for gold, which could be attributed to the sulfonate groups that constitute its chemical structure [49,50]. This could account for its existence in the AuNS lattice post-synthesis, as demonstrated by the elemental results (Figure 1E,F) and ^1^H-NMR analyses (Figure 1C), which are consistent with previous research [42,50,51].

The anisotropic AuNSs were characterized by a hybrid of two distinct UV-Vis absorbance peaks, corresponding to the plasmonic solid core (TLSPR spectra) and the plasmonic protuberant spikes (LLSPR spectra) [6,9,52]. The AuNS core acts as a nanoscale antenna, greatly enhancing the excitation cross-section and the electromagnetic field of the AuNS spike plasmons [6,9,53]. As a result of its hybrid shape, anisotropic AuNSs have a variety of energy states, with AuNS spikes having a higher energy level than the AuNS core [6,53,54]. The spikes have a higher energy state due to their increased surface area and localization of the electron flux from the core, which creates a strong electric field (lightning rod effect) [6,9,53].

During the AuNS bioassay synthesis, the relevance of NAD+ on the structure and activity of GalDH prompted us to co-immobilize GalDH (apoenzyme) and NAD+ (coenzyme) to the AuNSs. GalDH is a dimer dominated by a (β/α)_8_ barrel that permits NAD+ to bind and create an apoenzyme–coenzyme complex [44,55,56]. NAD+ acts as a conformation primer, stabilizing the oligomeric, catalytically active structure of the apoenzyme–coenzyme complex [57,58]. The apoenzyme–coenzyme complex, working on the C1 position of the sugar substrate, catalyzes the dehydrogenation of b-D-galactose pyranose to galactonate and NADH [59,60].

The colorimetric technique was divided into two phases: the generation of redox agents from the apoenzyme–coenzyme complex and the activation of morphological change in the AuNS structure by adding the detection solution [27]. The generation of redox agents on its own is insufficient to induce morphological changes, as shown in Figure 3 and previous studies [41,42,46]. In the presence of neo-formed NADH, the bioassay produced instantaneous monocolorimetric results (Figure 2A). Furthermore, in the H_2_O_2_-free assay, the rate of colorimetric signal generation was instantaneous; this phenomenon might be attributed to pH, temperature, or AuNP catalysis for the oxidative recycling of NADH to NAD [32,61,62]. In some instances, the addition of H_2_O_2_ to the bioassay resulted in better-localized surface plasmonic resonance (LSPR) band shifts and modest colorimetric signal generation rates (Figure 2B,C) [4,37,63]. As a result, the colorimetric signal generation rates increased as peroxide concentration increased (Figure 1D,E).

**Scheme 1:**
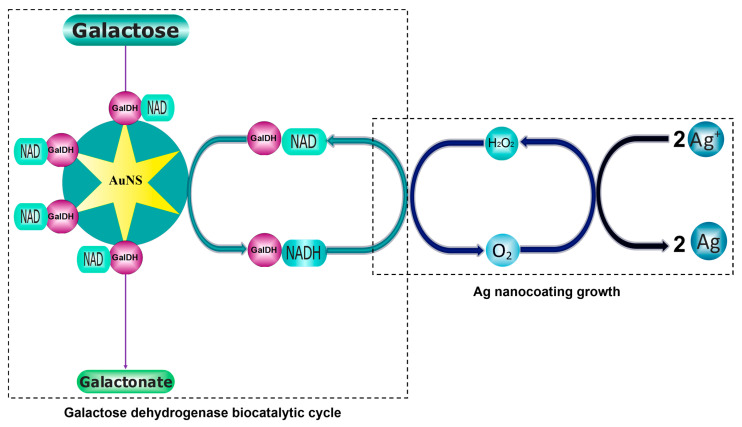
Proposed signal generation mechanism based on enzyme-H_2_O_2_-mediated crystal growth.

The assay generates an excess of NADH (reducing agent), and NADH is a probable primary reducing agent for Ag^+^ ions during the formation of the colorimetric signal [64]. Scheme 1 depicts our proposed signal generation technique, with NADH triggering Ag^+^ ions’ reduction on AuNSs and low H_2_O_2_ concentration causing AuNS remodeling into quasi-spherical AuNPs with enlarged core diameters, as supported by the TEM images (Figure 3) [34]. 

In the presence of the detection solution, however, H_2_O_2_ has the ability to oxidize both the AuNS and the neo-formed NADH, resulting in plausible morphological and colorimetric changes to AuNSs [34,65]. In simple terms, GalDH generates NADH, which reduces Ag+ ions to grow a Ag coating around the AuNS; furthermore, (i) at low concentrations of NADH and H_2_O_2_, the nucleation rate is slow, favoring the growth of a surface conformal Ag coating that induces a concentration-dependent blueshift in the AuNS’s LSPR and (ii) at high concentrations of NADH and H_2_O_2_, the fast crystal growth conditions favor Ag nanocrystal nucleation and less Ag is deposited on the AuNS, resulting in monocolorimetric signals (Figure 2). The plasmonic AuNS biosensor’s signal amplitude is determined by the rate of crystallization, which favors Ag^0^ coating growth on existing nanocrystals [34]. To validate this theory, the presence of an increase in the AuNP diameter in solutions containing a AuNS-PVP-GalDH biosensor after the biocatalytic reactions was observed using TEM images and Image-J software bundled with 64-bit Java 1.8.0_172 (Figure 4). These findings support the suggested mechanism whereby low H_2_O_2_ concentrations cause a slow rate of crystallization, which favors the growth of a Ag coating on Au seeds, and NADH is the primary Ag^+^-reducing agent.

When we compared standard chemistry to experimental work outcomes, we identified anomalies that did not totally support the proposed hypothesis. As a result, we offer theories and explanations for where these discrepancies emerge.

Discrepancy No. 1: gold nanoparticles and NAD(P)H reaction

According to standard redox chemistry, it is thermodynamically impossible for NAD(P)H to react with Au; hence, NAD(P)H has no ability to reduce Au or vice versa. In the presence of gold nanoparticles, however, the AuNS acts as a catalyst in the conversion of NAD(P)H to NAD(P).

Theory in response to Discrepancy No. 1

Using standard redox chemistry, Au^0^ must be etched, oxidized, or increased in the presence of NAD(P) and H_2_O_2_ because the reactions are thermodynamically feasible (Equations (1) and (2)).

(1) $$2Au+{3H}_{2}O_{2}+6H^{+}⟶{2Au}^{3+}+6H_{2}O$$

(2) $$2Au+3{NAD(P)}^{+}+3H^{+}⟶{2Au}^{3+}+3NAD(P)H$$

(3) $$2Au+3NAD(P)H+3H^{+}⟶2Au+3{NAD(P)}^{+}$$

**Figure 7:**
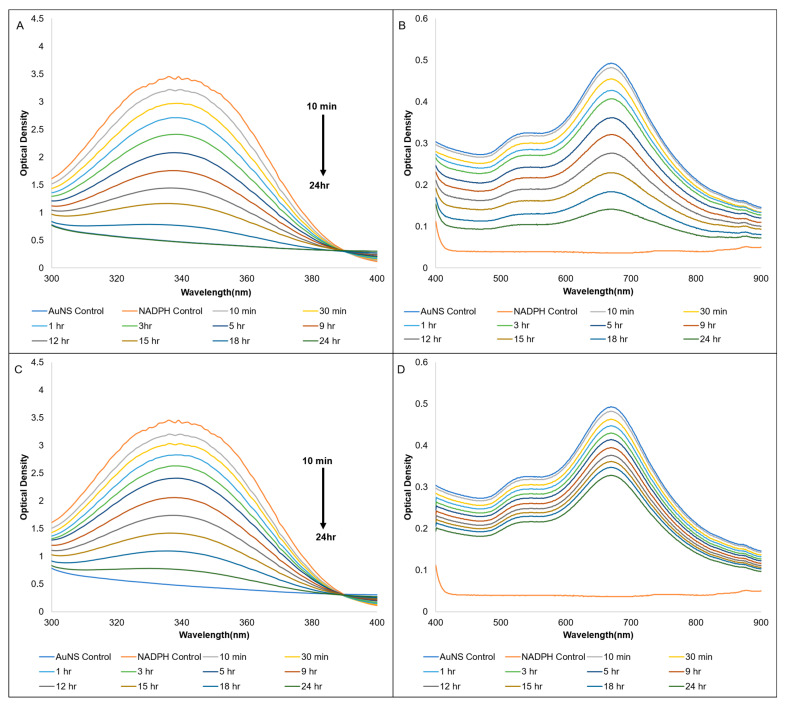
UV-Vis spectral profiles for the interaction between NAD(P)H and AuNSs over time in a water matrix. The NAD(P)H concentration and the AuNS UV-Vis spectral shifts are as follows: (**A**) 1 mM NAD(P)H and (**B**) AuNS spectra shift; (**C**) 0.05 mM NAD(P)H and (**D**) AuNS spectra shift.

Nonetheless, we employed spectrophotometry to follow the interaction of different NAD(P)H concentrations (Figure 7A ~1 mM; Figure 7C ~0.05 mM) with AuNSs during a 24 h period (Figure 6). Interestingly, the intensity of the 340 nm NAD(P)H absorption band dropped, while the intensity of the 260 nm NADP (+) absorption band increased (Figure 7A,C), verifying Equation (3). The AuNS spectral band, on the other hand, remained constant (Figure 7B,D), hinting that Au^0^ functions as a catalyst for NAD(P)H-to-NAD(P) oxidation (Equation (3)), a result similar to that found by Huang et al. (2005) [61]. As a result, this evidence reveals that NAD(P)H is transformed to NAD(P)+ on the surface of the AuNS, implying that the AuNS surface-catalyzes NAD(P)H.

Discrepancy No. 2: absence of plausible morphological or colorimetric change in AuNS

There is no plausible morphological or colorimetric change in the AuNS in the presence of reducing or oxidizing agents. This is only revealed after the detection solution (a metal ion and a base) is added; in this study, we utilized AgNO_3_ and NaOH (Figure 6). As a result, the chemistry involved in the generation of colorimetric signals is still unclear.

Theory in response to Discrepancy No. 2

A change in pH, as with many biocatalytic processes, has the potential to terminate the bioreaction. Furthermore, in alkali conditions, NaOH can activate H_2_O_2_, driving the equilibrium of acid dissociation (Equation (4)) and producing hydroperoxide anions [66].

(4) $$H_{2}O_{2}+{OH}^{-}⇌H_{2}O+{HOO}^{-}$$

In alkaline settings (pH 8–12) and at low temperatures (37 °C), hydrogen peroxide may quickly form hydroxide radicals (Equation (5)), which may explain the rapid biorecognition response in some cases and the requirement for alkaline conditions for AuNS signal production and remodeling.

(5) $$H_{2}O_{2}⟶2{OH}^{*}$$

As demonstrated by several of our findings, H_2_O_2_ is required for successful AuNP dissolution, and the AuNP dissolution rate is sensitive to H_2_O_2_ concentration, which is consistent with prior studies [67,68]. 

NAD(P)H has the potential to cause AuNP dissolution, as evidenced by some of our findings, and the AuNP dissolution rate is sensitive to NAD(P)H concentration, which is consistent with earlier studies [27]. In almost all aerobic systems, the addition of one electron to dioxygen (O_2_) nonenzymatically produces the superoxide anion (O_2_)^−^, which can become radicalized [69]. NAD(P)H can react with oxygen-centered radicals to create NAD(P)* radicals that are stable in an alkaline pH (Equation (6)), and they may be the ones triggering AuNS remodeling and mild dissolution [69,70,71].

(6) $$O_{2}+e^{-}⇢NAD\left(P\right)H+O_{2}^{-*}⇌NAD{\left(P\right)}^{*}\;\;\;$$

**Scheme 2:**
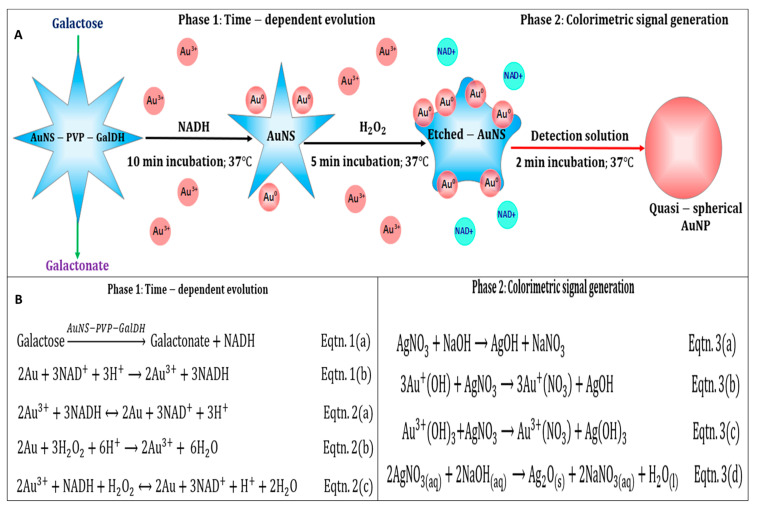
Proposed colorimetric signal generation and redox chemistry scheme. (**A**) AuNS remodeling schematic; (**B**) Proposed redox chemistry.

Scheme 2 displays our thought process prior to spectrophotometrically tracking the reaction and measuring the impact of detection solution on AuNS shape and colorimetric analysis. Using standard redox chemistry, there must be obvious spectral and colorimetric changes even in the absence of the detection solution in Scheme 2; nevertheless, nothing happens unless the detection solution is present.

The equations in Scheme 2B above are confirmed by our ICPS-MS data, which show no overall change in Au or Ag but an increase in Na, which may imply the likelihood of Ag coating growth, as suggested by several researchers, but we did not confirm this [34,72]. Furthermore, Ag is a lustrous metal, and it is possible to produce Ag/Ag_2_O nanoparticles of various shapes and sizes via plating or alloying with the Au in forming this colorimetric signal [73]. This is likely to have an impact on the plasmonic band and colorimetric signals that we receive in alkaline settings [74,75,76]. AuNPs, on the other hand, can form hydroxides on their surfaces, which can then react with the detection solution with a reduced likelihood of Equation (3b,c) [77]. In conclusion, we did not fully grasp how nanoparticles preserve their morphological integrity in the presence of oxidizing and reducing agents.

Overall, these findings demonstrate that the signal registered by the AuNS biosensor (transducer) together with the physico-chemical properties of the nanosensor, pH, temperature, detection solution concentration, H_2_O_2_ concentration, presence of capping agents, and presence of contaminants may impact the generation of the AuNS biosensor colorimetric signals. As a result, the findings suggest that, with appropriate assay tailoring, the AuNS-PVP-GalDH biosensor can be employed as both a qualitative (Figure 2A) and quantitative (Figure 2B) biosensor.

However, the AuNS-PVP-GalDH biosensor relied on the blue-shifted absorption band of the nanostar (690 nm) to quasi-spherical nanoparticles (540 nm) during NADH and H_2_O_2_ biocatalytic reactions (Figure 2). The bioassay has a potential downside when using a synthetic blood matrix because most synthetic blood components have significant absorption in the same range as the AuNPs [78,79,80]. In addition to AuNSs having a higher extinction coefficient when compared to other components, matrix dilution and blank subtraction approaches were also used to improve result interpretation and analysis [81,82].

Weng et al. (2019), on the other hand, described NADH-mediated suppression of H_2_O_2_-induced gold nanorod etching [28]. The bromine intermediates (Br_3_^−^ and Br_2_) from cetyltrimethylammonium bromide (CTAB) and 5-bromosalicylic acid (5-BrSA) used to etch gold nanorods were reduced and consumed in the presence of NADH, inhibiting the etching rate and resulting in a slight blue shift of the LPB. Maugeri et al. (2023) recently developed a unique photothermal-contrast method for detecting phenylalanine (Phe) in human blood using phenylalanine dehydrogenase (PDH), resulting in the formation of in situ AuNPs in the presence of neo-formed NADH and AuCl_4_^−^ [83]. Jafari et al. (2021) proposed a colorimetric-paper-based biosensor based on the PDH enzyme for highly sensitive and selective quantification of Phe, using neo-formed NADH, cationic dyes, and AuNPs as colorimetric mediators [84].

Our findings indicate that the NADH-H_2_O_2_-driven competitive assay biocatalytically drives AuNP growth, resulting in concentration-dependent morphological, spectrophotometric, and colorimetric changes. H_2_O_2_-mediated surface etching can be used to adjust the surface plasmon resonance of AuNSs by swiftly and sensitively etching the high-energy facets of the AuNS protrusions [6,53,54,85]. The NADH-catalyzed oxidation of Au^3+^ ions allowed for in situ AuNP development, which is consistent with the findings of Maugeri et al. (2023). The etched AuNSs serve as seeds for Au^0^ deposition, eventually leading to the biocatalytic development of larger-diameter quasi-spherical AuNPs [86]. Furthermore, AuNSs served as signal amplifiers and immobilization scaffolds in our biosensor design, eliminating the need for cationic dyes, as suggested by Jafari et al. (2021) [87,88,89]. 

## 5. Conclusions

In this study, AuNSs were employed to develop a multicolorimetric NADH biosensor based on simple and sensitive surface etching and the growth of AuNPs. In the presence of NADH, H_2_O_2_, and the detection solution, the AuNS-PVP-GalDH biosensor was etched into quasi-spherical nanostructures with increased core diameters. The etching and growth processes resulted in NADH-concentration-dependent colorimetric signals that were visible to the naked eye. Finally, the AuNS-PVP-GalDH assay offers a novel galactose plasmonic colorimetric detection method with remarkable sensitivity in simple and complex matrices. In addition, the NADH-H_2_O_2_-driven gold nanostar etching system provides a promising practical analytical target detection technique in resource-limited settings.

## Figures and Tables

**Table 1:** Experimental procedure for the galactose plasmonic colorimetric detection using AuNS-PVP-GalDH biosensor.

Sample	Control	1	2	3	4	5
Water	165 µL	148	138 µL	128 µL	118 µL	108 µL
10 mM Tris (pH 8.4)	15 µL	15 µL	15 µL	15 µL	15 µL	15 µL
AuNS-PVP-GalDH	20 µL	20 µL	20 µL	20 µL	20 µL	20 µL
2 mM galactose	0 µL	0 µL	5 µL	10 µL	15 µL	20 µL
10 min incubation						
H_2_O_2_	0 µL	0 µL	5 µL	10 µL	15 µL	20 µL
5 min incubation						
10 mM AgNO_3_	0 µL	2 µL	2 µL	2 µL	2 µL	2 µL
150 mM NaOH	0 µL	15 µL	15 µL	15 µL	15 µL	15 µL
2 min incubation						
Colorimetric signal generation						

## Data Availability

The supporting data are accessible from the Figshare repository. https://doi.org/10.6084/m9.figshare.23798394.v2 (accessed on 28 July 2023).
